# How effective are chest compressions when wearing mask? A randomised simulation study among first-year health care students during the COVID-19 pandemic

**DOI:** 10.1186/s12873-022-00636-2

**Published:** 2022-05-08

**Authors:** Bálint Bánfai, János Musch, József Betlehem, Emese Sánta, Balázs Horváth, Dániel Németh, Henrietta Bánfai-Csonka

**Affiliations:** 1 Faculty of Health Sciences, Institute of Emergency Care and Pedagogy of Health, University of Pécs, Vörösmarty street 4, 7621 Pécs, Hungary; 2 Faculty of Health Sciences Institute of Emergency Care and Pedagogy of Health, University of Pécs, Jókai Mór street 14, 9700 Szombathely, Hungary; 3 Clinical Centre, Department of Emergency Medicine, University of Pécs, Ifjúság street 13, 7624 Pécs, Hungary

**Keywords:** Chest compressions, COVID-19, Wearing mask, Fatigue, Health care students, Simulation

## Abstract

**Background:**

The resuscitation guidelines provided for the COVID-19 pandemic strongly recommended wearing personal protective equipment. The current study aimed to evaluate and compare the effectiveness of chest compressions and the level of fatigue while wearing two different types of mask (surgical vs. cloth).

**Methods:**

A randomized, non-inferiority, simulation study was conducted. Participants were randomised into two groups: surgical mask group (*n* = 108) and cloth mask group (*n* = 108). The effectiveness (depth and rate) of chest compressions was measured within a 2-min continuous chest-compression-only CPR session. Data were collected through an AMBU CPR Software, a questionnaire, recording vital parameters, and using Borg-scale related to fatigue (before and after the simulation). For further analysis the 2-min session was segmented into 30-s intervals.

**Results:**

Two hundred sixteen first-year health care students participated in our study. No significant difference was measured between the surgical mask and cloth mask groups in chest compression depth (44.49 ± 10.03 mm vs. 45.77 ± 10.77 mm), rate (113.34 ± 17.76/min vs. 111.23 ± 17.51/min), and the level of fatigue (5.72 ± 1.69 vs. 5.56 ± 1.67) (*p* > 0.05 in every cases). Significant decrease was found in chest compression depth between the first 30-s interval and the second, third, and fourth intervals (*p* < 0.01).

**Conclusion:**

The effectiveness of chest compressions (depth and rate) was non-inferior when wearing cloth mask compared to wearing surgical mask. However, the effectiveness of chest compressions decreased significantly in both groups during the 2-min chest-compression-only CPR session and did not reach the appropriate chest compression depth range recommended by the ERC.

## Background

Out-of-hospital cardiac arrest (OHCA) is a global health problem. In Europe and in the US, hundreds of thousands of people die annually due to sudden cardiac arrest (SCA) [1, 2]. In Hungary, 45–50,000 people die annually because of different cardiac reasons [3]. The immediate recognition of cardiac arrest, activation of the emergency response system, early cardiopulmonary resuscitation (CPR), and defibrillation can improve the survival rates after SCA [4]. The coronavirus disease (COVID-19) emerged in Wuhan in late 2019 and rapidly caused a worldwide pandemic [5]. The disease is caused by the severe acute respiratory syndrome coronavirus-2 (SARS-CoV-2) and is highly contagious. Evidence on the main routes of transmission remains limited and evolving, and influenced by several factors [6]. During the pandemic, the number of OHCA increased in several European countries but the rate of first responders providing help decreased [7–10]. In Sweden, 10% of OHCA patients were affected during the COVID-19 pandemic [11].

Introducing CPR training at schools is an effective way to improve the community response after OHCA. The KIDS SAVE LIVES campaign has been successfully implemented in several countries worldwide [12]. However, minimal CPR training is provided in higher education. Mandatory CPR courses would be necessary in higher education in every field, especially for health care students [13]. In Hungary, CPR training is compulsory for all medical and health care students, however, the competencies acquired before graduation are limited [14]. CPR competencies and knowledge of cardiac arrest vary widely across Europe [15].

High quality chest compressions (CC) without unjustified interruptions constitute the key element of effective CPR [16, 17]. The quality of CC can be affected by several factors (e.g. gender, body weight, general fitness, known diseases, etc.) [18, 19]. Rescuers’ fatigue can decrease the effectiveness of CC [20]. Based on the current guidelines of the European Resuscitation Council (ERC), changing the rescuers every two minutes is recommended [16]. COVID-19 has a high impact on real CPR as well as on training. ERC reacted quickly to the pandemic situation and prepared new guidelines. The focus of the new recommendations is to decrease the risk of contamination for the first responder. Therefore, wearing personal protective equipment (PPE) is strongly recommended [21]. However, a previous systematic review and meta-analysis showed some negative effects when wearing PPE during CPR [22].

The aims of the current study were to compare the effectiveness of CC and the level of fatigue wearing two different types of masks (surgical mask group [SMG] vs. cloth mask group [CMG]) among first-year health care students in a 2-min long continuous chest compression-only CPR (CCO-CPR) scenario. We hypothesized that CCO-CPR performace with wearing cloth mask is non-inferior to wearing surgical mask.

## Methods

A randomised simulation non-inferiority study was conducted to investigate the effects of wearing two different types of mask (SMG vs. CMG) and gloves while performing CCO-CPR among health care students. The reasons for involving surgical masks and cloth masks in our study were: (1) during the pandemic the majority of the Hungarian population wear these types of masks and the ratio of wearing higher quality PPE (e.g. N95 mask) is rare in the general lay population; and (2) no previous studies have investigated the effects of wearing cloth mask during CPR. The following consumer-grade face masks were used by the participants in CMG: a 2-layer, washable, polyester mask with ear loops and without nose bridge (Rovitex Hungária Kft). In the SMG, medical procedure masks with elastic ear loops and aluminium nose bridge were used by the students (LND Pharma Kft.).

### Participants

First-year health care students (dietetics, nursing, paramedic and physiotherapy) from the University of Pécs Faculty of Health Sciences participated in our study between September and October 2020. All first year students were informed about the study through a written form and were asked to contact the trial manager if they were willing to participate. Students were recruited for the study after their “First aid and resuscitation” course. This subject, taught to every health care student in the first semester of their university studies, contains two hours of theory and two hours of practice about adult basic life support (BLS). BLS content was based on the current ERC guidelines, including COVID-19 recommendations [16, 21]. All students were alternately allocated to the two different groups (SMG and CMG). Simple randomization method was used by computer generated random numbers when participants arrived (even – SMG, odd – CMG). The participants were blinded to their assigned groups until they were started the measurement. In addition, they were also blinded to the purpose of the study until the end of the study.

### Study design and data collection

Data collection consisted of three different parts: (1) self-administered questionnaire; (2) vital parameters in relationship with fatigue; (3) effectiveness of CC through AMBU CPR Software (AMBU A/S, Baltorpbakken 13, DK 2750 Ballerup, Denmark).

The questionnaire contained the following data: type of mask, sex, age, smoking habit, known cardiovascular and/or respiratory disease, and prior first aid training. In addition, the questionnaire contained the Borg-scale [23] to collect information about the subjective feeling of fatigue before and after the 2-min continuous CCO-CPR session (1–10 points; 1 = does not disturb it, 10 = makes it very difficult).

Before starting the CCO-CPR session, body weight and body height were measured and body mass index (BMI) was calculated as potential influencing factors of CC quality.

The primary outcome of our study was to measure and compare CC’s effectiveness (depths and rate) in the two different groups. To evaluate students’ CCO-CPR performance, the AMBU CPR Software was used and the depth and rate of CC were detected during a 2-min-long continuous session. During the analysis, the 2-min period was segmented into 30-s intervals to evaluate data more precisely. The data of the software (CC depth and rate) were blinded for the participants during performing the CCO-CPR session so they did not receive any type of feedback on their performance.

The secondary outcome of our study was to evaluate and compare participants’ fatigue in the two different groups. The blood-pressure (BP), oxygen-saturation (SpO_2_), heart-rate/pulse (HR/P), and respiratory-rate (RR) were measured immediately before and after the continuous 2-min-long CCO-CPR session. To measure BP, SpO_2_ and HR/P, certified devices were used and RR was measured by inspection. All vital parameters were measured by an experienced paramedic. The subjective feeling of fatigue was measured by the Borg-scale as mentioned above.

All these situations were led by paramedics experienced in CPR training.

### Sample size calculation

The sample size was based on expected differences in CC depth and rate. The mean standard deviation (SD) was for CC depth of 10.5 mm and for CC rate of 17.6/min. With a power of 80% and an alpha error probability of 5%, the estimated sample size was 110 for CC depth (non-inferiority margin of 3 mm) and 153 for CC rate (non-inferiority margin of 4/min). To cover both main data, we decided for a minimum sample size of 153 (minimum 77/group). We tried to recruit as many first year health care students as possible.

### Statistical analysis

Statistical analysis was conducted using SPSS 24.0 (Statistics Package for Social Sciences, Chicago, IL, USA) statistical software. Descriptive statistics were performed (percentage, mean, standard deviation) to describe the sample. Study parameters were assessed for normality by using Shapiro–Wilk test which indicated normal distribution. A t-test was used to evaluate the association between effectiveness of CC (depth and rate), fatigue (vital parameters and Borg-scale), and the type of mask. A two-tailed p-value less than 0.05 was considered to be statistically significant.

## Results

**Fig. 1:**
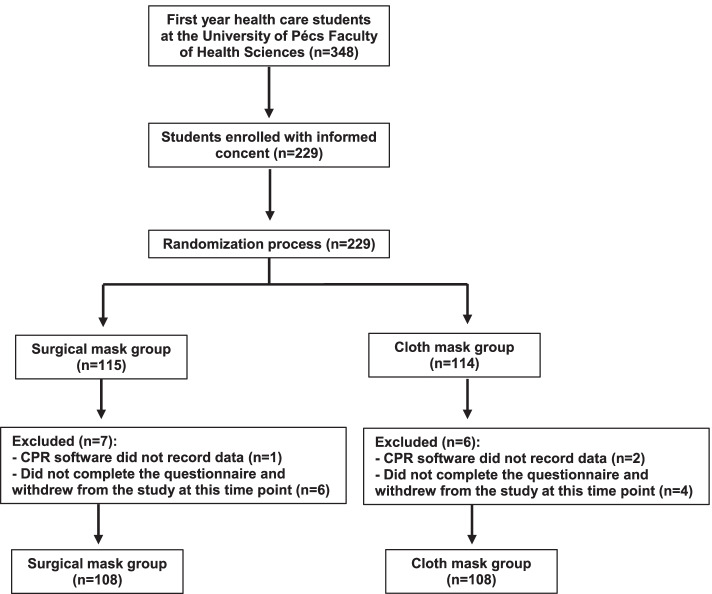
Study flow-chart

A total of 229 students were recruited for the study. Thirteen of the participants were excluded because of the exclusion criteria (in three cases CPR software did not record data and ten students did not complete the questionnaire and withdrew from the study before the CCO-CPR examination). After exclusions, the total number of participants was 216 (*n* = 216) (Fig. 1). After randomization, 108 students were included in the SMG and 108 students in the CMG. The main characteristics of the participants are shown in Table 1.

**Table 1:** The main characteristics of the students (*n* = 216)

		**Type of mask**	
		**SMG (*n*** **= 108)**	**CMG (*n* = 108)**
**Sex**	*Man*	11.1% (12)	14.8% (16)
	*Woman*	88.9% (96)	85.2% (92)
**Mean age**		19.44 ± 1.654	19.19 ± 1.226
**Mean body weight**		63.01 ± 10.763	64.11 ± 14.411
**Mean body heigh**		166.88 ± 8.805	170.31 ± 8.691
**Mean BMI**		22.039 ± 2.968	22.024 ± 4.248
**Smoking habit**	*Yes*	13% (14)	11.1% (12)
	*No*	87% (94)	88.9% (96)
**Known cardiovascular and/or respiratory disease**	*Yes*	7.4% (8)	4.6% (5)
	*No*	92.6% (100)	95.4% (103)
**Prior resuscitation training**	*Yes*	54.6% (59)	61.1% (66)
	*No*	45.4% (49)	38.9% (42)

Data are normally distributed

Categorical variables are presented in percentages and absolute numbers in parentheses. Continuous variables are presented as mean and standard deviation

*SMG *Surgical mask group, *CMG* Cloth mask group, *BMI* Body Mass Index

Age, body weight, body heigh, BMI, smoking habit, known diseases and prior training were similar between the two groups. Most of the participants were female.

### Primary outcome—Effectiveness of chest compressions

The effectiveness of CC was measured using the AMBU CPR software. The comparison of SMG vs. CMG are demonstrated in Table 2.

**Table 2:** The effectiveness of chest compressions performed by the students (*n* = 216)

		**Type of mask**		***p-value***	**Mean difference [CI 95%]**
		**SMG **(***n*** **= 108)**	**CMG **(***n*** **= 108)**		
**Mean depth of CC (mm)**	*0–29.9 s*	48,26 ± 14.15	48.55 ± 9.97	*0.86*	-0.27 [-3.6, 2.9]
	*30–59.9 s*	45.14 ± 10.88^*^	46.19 ± 10.71^*^	*0.47*	-1.1 [-3.9, 1.8]
	*60–89.9 s*	42.88 ± 10.08^*^	44.76 ± 11.09^*^	*0.19*	-1.9 [-4.7, 0.9]
	*90–120 s*	41.69 ± 10.41^*^	43.77 ± 14.72^*^	*0.23*	-2.1 [-5.5, 1.3]
	*2 min in total*	44.49 ± 10.03	45.77 ± 10.97	*0.41*	-1.2 [-4, 1.6]
**Mean rate of CC (/min)**	*0–29.9 s*	113.28 ± 16.17	110.96 ± 15.94	*0.29*	2.3 [-1.9, 6.6]
	*30–59.9 s*	113.57 ± 17.97	111.29 ± 18.00	*0.35*	2.2 [-2.5, 7.1]
	*60–89.9 s*	113.56 ± 18.48	112.04 ± 18.00	*0.54*	1.5 [-3.4, 6.4]
	*90–120 s*	112.94 ± 20.07	110.63 ± 20.16	*0.39*	2.3 [-3.1, 7.7]
	*2 min in total*	113.34 ± 17.76	111.23 ± 17.51	*0.38*	2.1 [-2.6, 6.8]

Data are normally distributed

*SMG* Surgical mask group, *CMG* Cloth mask group, *CC* chest-compression, *CI* Confidence interval

Results are presented as mean and standard deviation. The 2-min continuous chest-compression session was segmented into 30 s intervals. Differences between the SMG and CMG related to CC’ depth and rate were measured using t-test

^*^p < 0.05 compared to the first interval (t-test was used)

In the primary outcome, there was no significant difference between the mean CC depth (*p* = 0.41) and CC rate (*p* = 0.38) of the SMG and CMG. The depth of CC decreased significantly after the first 30-s interval (*p* < 0.01) (both in SMG and CMG) compared to the second, third, and fourth 30-s intervals but the CC rate did not change significantly over time. There was no difference detected between SMG and CMG during the 30-s intervals. The differences in depth and rate of CC between 30-s intervals are shown in Table 2.

### Secondary outcome—Fatigue during performing chest compression

Vital parameters and subjective fatigue (based on the Borg-scale) are shown in Table 3.

**Table 3:** Vital parameters and subjective fatigue (based on the modified Borg-scale) (*n* = 216)

		**Type of mask**		***p-value***	**Mean difference [CI 95%]**
		**SMG (*n*** **= 108)**	**CMG (*n*** **= 108)**		
**Mean blood pressure (Systolic) (Hgmm)**	*Before*	121.66 ± 13.52	121.10 ± 14.25	*0.77*	0.6 [-3.1, 4.2]
	*After*	128.55 ± 15.12	127.07 ± 15.41	*0.48*	1.4 [-2.6, 5.6]
**Mean blood pressure (Diastolic) (Hgmm)**	*Before*	74.20 ± 12.78	73.86 ± 9.20	*0.82*	0.3 [-2.6, 3.3]
	*After*	72.05 ± 9.55	72.65 ± 8.78	*0.63*	-0.6 [-3.1, 1.9]
**Mean heart rate/pulse (/min)**	*Before*	90.26 ± 19.05	90.12 ± 19.17	*0.96*	0.1 [-4.9, 5.2]
	*After*	106.35 ± 21.87	106.61 ± 23.94	*0.93*	-0.2 [-6.4, 5.9]
**Mean oxygen-saturation (%)**	*Before*	97.88 ± 1.60	97.82 ± 1.50	*0.79*	0.1 [-0.4, 0.5]
	*After*	97.16 ± 1.91	97.16 ± 1.92	*0.99*	0 [-0.5, 0.5]
**Mean respiratory-rate (/min)**	*Before*	15.39 ± 2.51	15.19 ± 2.67	*0.58*	0.2 [-0.5, 0.9]
	*After*	21.08 ± 3.53	21.01 ± 3.83	*0.88*	0.1 [-0.9, 1.1]
**Mean Borg-scale (1–10)**	*Before*	4.45 ± 1.76	4.26 ± 1.69	*0.19*	0.3 [-0.2, 0.7]
	*After*	5.72 ± 1.69^*^	5.56 ± 1.67^*^	*0.49*	0.2 [-0.3, 0.6]

Data are normally distributed

Numbers are demonstrated as mean and standard deviation. Differences between the SMG and CMG related to the fatigue (vital parameters, Borg-scale) were measured using t-test

*SMG* Surgical mask group, *CMG* Cloth mask group, *CI* Confidence interval

^*^p < 0.01 compared to the Borg-scale after the 2-min continuous chest-compression session (t-test was used)

In the secondary outcomes, no significant difference was measured in changes the vital parameters and the Borg-scale between SMG and CMG. Borg-scale increased significantly in both of the groups (SMG and CMG) after the 2-min continuous CCO-CPR session (*p* < 0.01). No significant difference was detected in the Borg-scale between SMG and CMG before (*p* = 0.19) and after (*p* = 0.49) the 2-min continuous CCO-CPR session.

After performing CC, systolic BP increased in every groups (*p* < 0.01) but diastolic BP did not change; RR and HR/P increased (*p* < 0.01) but SpO_2_ decreased (*p* < 0.01).

## Discussion

The main aims of our non-inferiority study were to compare the effectiveness of CC when wearing different types of masks (surgical vs. cloth masks) and to measure the level of fatigue during a 2-min long continuous CCO-CPR session among first-year health care students. We found no differences between wearing surgical or cloth mask on CCO-CPR effectiveness and the level of fatigue. The strength of our study is that to the best of our knowledge, no previous study investigated and compared the effectiveness of CCO-CPR when wearing only lower quality masks (surgical and cloth masks that are available for general population) and not high filtered PPE (e.g. N95). In addition, we investigated a wide range of first-year health care students (dietetics, nursing, paramedic, and physiotherapy). The participants of our study are considered laypeople at this stage of their studies. Previous studies primarily investigated graduates from health care programs or health care students immediately before their graduation [13–15, 24–26].

During the COVID-19 pandemic, wearing masks and other available types of PPE has been strongly recommended during CPR [21] because CC can be an aerosol-generating procedure (AGP) [21, 27]. A recent study demonstrated that both surgical and cotton mask seem to be ineffective in preventing the dissemination of SARS-CoV-2 through coughs of infected patients with COVID-19 [28]. Another study revealed that the fitted filtration efficiency (FFE) of different cloth masks that are available for the public is nearly equivalent to or better than e.g. surgical masks in many cases [29]. Despite the previously mentioned results, cloth and/or surgical masks together with other strategies (e.g. social distancing and regular hand washing) can provide some reduction of contamination from expiratory particles [30, 31]. Prior studies primarily focused on a wider range of PPEs used during professional patient care and not lay-CPR [32–35].

In our study, there were no significant differences detected between the performance of SMG and CMG. The effectiveness of CC decreased significantly in both groups during the 2-min CCO-CPR session. Some vital parameters (BP, HR/P, RR) increased significantly and SpO_2_decreased significantly after the 2-min continuous CC session. Subjective fatigue based on the modified Borg-scale increased after performing CCs but it did not influence the performance of CCO-CPR (neither depth nor rate of CC). A recent study showed that health care professionals wearing N95 mask showed lower quality of CPR and fatigue in a shorter time period than participants wearing surgical masks [33]. In another recent study, which demonstrated results about medical students who wore full PPE, CC depth decreased after the first minute significantly in both groups with or without feedback device. The CC rate was better in the group using a feedback device [34]. Conversely, a very recent study demonstrated that there was no differences between the CPR performance of emergency medical service providers who wore and did not wear different types of masks [35]. However, owing to the current national regulations, wearing some types of PPE (at least a face mask) in the streets or at workplaces is mandatory, so in the case of OHCA in lay CPR there should be no delay in wearing it. Therefore, based on this limited evidence, wearing PPE is still recommended to protect bystanders from contamination [21, 27, 36].

In our study, there was no significant difference between the mean CC depth and rate in SMG and CMG. As previous studies have shown, there are several factors that influence the effectiveness of CC [17–20]. Students in our study performed poorer CC depth (44.49 ± 10.03 mm in SMG and 45.77 ± 10.97 mm in CMG) compared to the 5–6 cm depth recommended by the current ERC guidelines [16]. The CC rate (113.34 ± 17.76 in SMG and 111.23 ± 17.51 in CMG) was within the recommended interval (100–120/min). In contrast, a study from Norway reported data about higher quality CPR without wearing PPE, which supports the current recommendations among final year nursing students [37]. However, based on a previous analysis, CC depths between 40 and 55 mm with a peak at 46 mm were associated with highest survival rates [38]. During our investigation, CC depth decreased and CC rate increased over time, similar to the findings of a previous study [39]. The CC rate did not change significantly during the investigation and CC depth decreased significantly after the first 30-s interval compared with the second, third and fourth intervals. This result is in contrast with prior investigations among paramedics, which did not show deterioration in CC quality within the first 2 min [24, 25]. However, some other studies reported data about rapid deterioration in the quality of CPR after one minute, similarly our findings [40]. Therefore, changing the rescuer more frequently than every 2 min should be considered.

### Limitations

Despite promising results, our study has several limitations. Our study might not be representative of the whole population of health care students nor the entire population in Hungary; thus, the effects of wearing surgical and/or cloth mask remains unknown. Further, distribution of sex was not equal (the majority of respondents were female). However, this ratio is general and acceptable in the field of health care in higher education in Hungary. In our study, simple randomization method was used which can cause some biases in allocation of participants. Therefore, permuted block randomization could be more reliable. Since only a 2-min-long continuous CCO-CPR session was measured, we do not have information about further and later effects (e.g. higher level of fatigue, lack of other rescuer, not optimal surface, etc.). However, we chose this time interval because in the current ERC guidelines changing the rescuer is recommended every 2 min. This study did not establish a control group to measure the effectiveness of students’ CC without wearing masks, which would be a useful comparison for further analysis. Nonetheless, wearing a mask is strongly recommended during the pandemic. While a CPR manikin was used in our study, the actual CPR effectiveness of these students in a real cardiac arrest situation remains unknown. We did not use any feedback device which could improve the quality.

## Conclusion

In conclusion, according to the results of our study, the effectiveness of CC was non-inferior when wearing cloth mask compared to wearing surgical mask. However, the effectiveness of CC decreased significantly in both groups during the 2-min CCO-CPR session and did not reach the appropriate CC depth range recommended by the ERC. Further studies are needed to measure and improve CC quality when wearing mask.

## Data Availability

The datasets used and/or analysed during the current study are available from the corresponding author on reasonable request.

## Abbreviations

OHCA: Out-of-Hospital Cardiac Arrest
CPR: Cardiopulmonary Resuscitation
COVID-19: Coronavirus Disease
SARS-CoV-2: Severe Acute Respiratory Syndrome Coronavirus-2
CC: Chest Compression
ERC: European Resuscitation Council
PPE: Personal Protective Equipment
CCO-CPR: Chest Compression Only Cardiopulmonary Resuscitation
SMG: Surgical Mask Group
CMG: Cloth Mask Group
BP: Blood Pressure
HR/P: Heart Rate/Pulse
SpO_2_: Oxygen Saturation
RR: Respiratory Rate
CI: Confidence Interval
